# Occupational Exposure to Endotoxin along a Municipal Scale Fecal Sludge Collection and Resource Recovery Process in Kigali, Rwanda

**DOI:** 10.3390/ijerph16234740

**Published:** 2019-11-27

**Authors:** Rachel Sklar, Zeyi Zhou, Marley Zalay, Ashley Muspratt, S. Katharine Hammond

**Affiliations:** 1 Environmental Health Sciences Division, School of Public Health, University of California, Berkeley, CA 94720, USA; zeyi_zhou@berkeley.edu (Z.Z.); hammondk@berkeley.edu (S.K.H.); 2 Office of Environmental Health Hazard Assessment, Oakland, CA 94612, USA; marley.zalay@oehha.ca.gov; 3 Independent Consultant, Northampton, MA 01060, USA; murray.ash@gmail.com

**Keywords:** occupational exposure assessment, endotoxin, fecal sludge management, sanitation, Rwanda, mathematical modeling

## Abstract

*Background*: Little is known about occupational exposures that occur along fecal sludge collection and resource recovery processes. This study characterizes inhaled endotoxin exposure to workers of a municipal scale fecal sludge-to-fuel processes in Kigali, Rwanda. *Methods*: Forty-two task-based air samples were collected from workers in five tasks along the fecal sludge collection and resource recovery process. Samples were processed for endotoxin using the limulus amebocyte lysate (LAL) test. To account for exposure variability and compare measured concentrations to established exposure limits, we used Monte Carlo modeling methods to construct distributions representing full eight-hour (8-h) exposures to endotoxin across eight exposure scenarios. *Results*: Geometric mean (GM) endotoxin concentrations in task-based samples ranged from 11–3700 EU/m^3^ with exposure concentrations increasing as the dryness of the fecal sludge increased through processing. The thermal dryer task had the highest endotoxin concentrations (GM = 3700 EU/m^3^) and the inlet task had the lowest (GM = 11 EU/m^3^). The geometric means (GM) of modeled 8-h exposure concentrations were between 6.7–960 EU/m^3^ and highest for scenarios which included the thermal dryer task in the exposure scenario. *Conclusions*: Our data suggest the importance of including worker exposure considerations in the design of nascent fecal sludge management processes. The methods used in this study combine workplace sampling with stochastic modeling and are useful for exposure assessment in resource constrained contexts.

## 1. Introduction

Onsite sanitation systems such as pit latrines or septic tanks currently serve more than 2.7 billion people globally and this number is expected to be as high as 4.9 billion by 2030 [1]. There is a growing consensus that in rapidly urbanizing contexts, such non-sewered sanitation systems may be more viable than centralized sewer systems which require high capital investment, operational expertise, and a dependence on scarce resources such as water and energy [2].

Fecal sludge, the high-density waste stream that arises from onsite systems, contains infectious pathogens which must be inactivated or removed in order to protect human health. Given the high concentration of solids and contaminants in fecal sludge waste streams, conventional wastewater treatment processes are neither effective or affordable for treating fecal sludge in resource strapped contexts [3]. As it stands, wastewater treatment systems which are built in low-resource contexts are often abandoned because of factors such as inadequate fecal sludge management infrastructure and generally poor operation and maintenance strategies [4].

A promising alternative for managing fecal sludge waste streams is through resource recovery systems which function by recycling fecal sludge into valuable products such as compost, biogas, solid fuel, or protein for animal feed [5,6]. The generation and sale of waste-derived products can be used to offset the high cost of sanitation service delivery and lower the cost of sanitation service delivery [5,7]. As such, an increasing number of technical innovations are emerging to close gaps in sanitation coverage as well improve the access to resources in low-income contexts [8,9,10].

While resource recovery systems promote obvious beneficial health outcomes for communities, less is known about the potential health risks for workers in resource recovery processes, who may come into close contact with human waste containing numerous chemical and microbiological hazards [11]. In 1989 the World Health Organization (WHO) released guidelines for the safe use of wastewater, excreta, and gray water. Updated in 2006, the guidelines recommend management practices to minimize health risks associated with the use of resource recovery end products. The international labor organization (ILO) also released a hazard sheet on risks associated with wastewater handling in 2012 [12]. However, both guidelines are far from comprehensive, with a focus on the exposure route of ingestion rather than the less-studied inhalation route. This is despite a well-established and growing body of literature characterizing hazards associated with the dusts and aerosols generated during the handling and treatment of sewage and fecal sludge [13].

Endotoxin is widely believed to play a causal role in the development of health effects resulting from exposure to organic dusts [14,15,16,17]. Endotoxin exposure has been associated with health effects in workers of sewage treatment plants [18,19,20], solid waste management processes [21,22,23], and other occupational settings that involve handling of organic materials—cotton textile industry [24], livestock farming [17], and composting [25]. Acute endotoxin inhalation exposures in workplaces are associated with chest tightness, bronchoconstriction, fever, cough, headache, nose and throat irritation, chest tightness, acute airway flow restriction, and inflammation [26,27]. Chronic exposures to inhaled endotoxin are associated with restrictive respiratory diseases such as asthma and chronic obstructive pulmonary disorder [28,29].

There is a mounting need to assess and characterize the exposure risks to workers of resource recovery processes. Failing to do so during the research and development stage of these nascent systems can result in systems which are designed to provide benefit to communities at the expense of worker health.

The aim of the present work is to characterize endotoxin exposure to workers at each stage in a resource recovery process which converts fecal sludge into solid fuel. The results presented in this study are based on samples collected of individuals working at a municipal scale fecal sludge-to-fuel operation in Kigali, Rwanda.

## 2. Materials and Methods

### 2.1. Study Site

All samples were collected in the city of Kigali which is currently home to 1.3 million people. Kigali has no system of sewers. Instead, households rely on decentralized, on-site sanitation systems. The vast majority, 95% of Kigali residents, use pit latrines as their primary form of sanitation [30]. When these facilities fill, the waste is removed by service providers who provide the emptying and transport service to Nduba Hill, Kigali’s landfill disposal site for both solid and liquid waste.

In 2015, the City of Kigali and a private American firm commissioned a pilot municipal scale fecal sludge-to-fuel plant situated at the Nduba dump site. Fecal sludge was emptied from households with full pits or septic tanks, transported to the Nduba resource recovery plant, and converted into solid fuel for use in cement production and other industry.

### 2.2. Process Description

**Figure 1:**
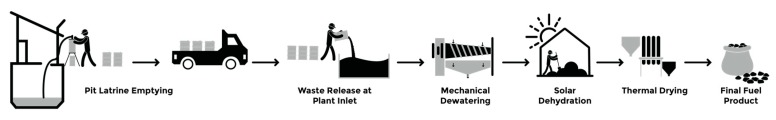
Schematic of waste collection, transport, and resource recovery/transformation to fuel.

We examine endotoxin exposure to workers in each of the following tasks of a municipal scale fecal sludge collection and resource recovery process (Figure 1):

- Pit Latrine Emptying and Waste Transport: The emptying process started with three preparatory steps: (1) Breaking the concrete slab, or floor of the latrine; (2) fluidizing the sludge with water and mixing it with a rod to facilitate extraction; and (3) removing trash using a “fishing” method in which a long hook is used to remove pieces of trash from the sludge filled pit (households often dispose of trash in pit latrines when solid waste management services are too expensive or out of reach) and place them into barrels for subsequent disposal. Fishing was done periodically during the emptying process to prevent solid waste from clogging the vacuum pump hose. Sludge emptied from pit latrines was on average 9% total solids. Fecal sludge accumulated in a pit latrine was emptied by a worker using a portable vacuum system to empty sludge into barrels which are sealed and then transported by truck to the fecal sludge-to-fuel plant.
- Waste release at plant inlet: During the waste release at the inlet of the plant, workers unloaded the barrels from the truck and poured sludge into the plant inlet basin directly from the barrels.
- Mechanical dewatering: The sludge was conveyed from the inlet by gravity to an MS-80 mechanical dewatering machine consisting of a rotating membrane and a screw auger. In this physical separation process, sludge was conveyed through a screw auger enclosed by an outer screen. Dewatering was accomplished as gravity drainage allows the filtrate to fall out of the solution and the solids are compressed and dewatered as the screw diameter decreases toward the outlet of the pipe. The MS80 operator was responsible for periodic monitoring of the machine as well as routine sample collection of the influent and effluent. The operator was also in charge of routinely opening the MS80 machine to prevent overflows as well as clean the machine before the task end. The result of this step was sludge “cake” which was approximately 25% total solids and a liquid effluent with significantly reduced treatment requirements. Effluent from this stage was treated for reuse in agriculture while the cake was transported to the greenhouses for solar dehydration.
- Solar dehydration: Workers spread the sludge cake from mechanical dewatering on the floor of a solar greenhouse system for evaporative drying. Here, the workers manually turned and mixed the material over a period of five to seven days depending on the ambient temperature and humidity, until the material reaches an average of 80% total solids.
- Thermal drying: In this task, a worker loaded sludge from the greenhouse into a vertical drum dryer in order to remove residual moisture, achieve ≥95% total solids, and deactivate and/or eliminate all pathogenic material. At the end of each rotating dryer cycle, a blower fan conveyed the final fuel product from the inlet of the drum to the outlet and a worker filled bags with the final fuel product.

### 2.3. Sampling Strategy

Two cross sectional sampling campaigns were carried out between July–August 2016 and May–August 2017. During the first campaign, area air samples (*N* = 20) were collected during the pit latrine emptying process. During the second campaign, personal air samples (*N* = 42) were collected from the personal breathing zone of 13 workers performing five tasks in the waste collection and resource recovery process: (1) Septic tank/pit latrine emptying; (2) truck waste release at the inlet of the fecal sludge-to-fuel plant; (3) mechanical dewatering machine operation; (4) solar drying in greenhouses; (5) thermal drying and fuel packaging.

For both area and personal samples, air was sampled onto a 37 mm Teflon filter (Pall Life Sciences, NY, USA) with an AirCheck TOUCH sampling pump (SKC Inc., PA, USA) at flow rate between 1.0–3.5 L/min.

Workers wore the personal air monitors for the duration of each task measured, including breaks. All workers included in this study wore their normal personal protective equipment provided by their employer during this sampling campaign. We considered potential candidates for inclusion of all workers performing jobs in the waste collection and transformation process who were over the ages of 18 and able to wear the samplers for the entire shift. All workers performed a single task during the sampling period. All workers consented to participating in the study, and all study protocols were approved by the UC Berkeley Committee for Protection of Human Subjects prior to the conduct of this research.

Data on the total solids by mass (TS) of the waste handled at each stage in the following process were sourced from the Nduba plant operations log.

### 2.4. Endotoxin Sample Analysis

The kinetic Limulus assay with resistant-parallel-line estimation (KLARE) method was used to determine the presence of endotoxin in all area and personal air samples collected onto Teflon filters [31]. Endotoxin was extracted by sonication with 5 mL of triethylamine phosphate buffer for one hour, and the sample concentrations were evaluated using the kinetic chromogenic LAL assay (Lonza Inc., Walkersville, MD, USA). Samples were mixed with lysate and loaded onto 96-well plates. Optical density was monitored over time by an absorbance microplate reader (Biotek Instruments, Winooski, VT, USA). Sample concentrations were first determined in endotoxin units (EU), then converted to EU/m^3^ by incorporating information on flow rate and volume during sample collection. The limit of detection per filter was 0.15 EU/mL.

### 2.5. Eight-Hour TWA Modeling and Comparison to OELs

Most countries lack an occupational standard for inhaled endotoxin exposure because of the absence of a standardized endotoxin detection protocol [32]. In this study we use the health-based OEL of 90 EU/m^3^ for an eight-hour (8-h) time-weighted average (TWA) proposed by the Health Council for the Netherlands (DECOS) as a benchmark to compare the exposure concentrations that we measured in this study [33]. The task-based concentration measurements were collected over tasks with durations shorter than 8 h. However, the OEL is in terms of an 8-h TWA. Thus, to compare the task-based concentrations to the 8-h TWA standard, we used our measured concentration data to model 8-h TWAs over different exposure scenarios. Because individual tasks are not completed for a full 8-h duration, we created exposure scenarios which represent different rotation schedule (a series different tasks performed throughout the 8-h workday). Six of the eight scenarios are rotation schedules for the resource recovery team working on the premises of the fecal sludge-to-fuel plant and two of the scenarios represents the waste collection team performing the pit latrine emptying tasks over an 8-h period, reflecting separation between these teams and their tasks. Six of the scenarios were designed to minimize the time spent in the thermal dryer as the measured endotoxin concentrations in this task were considerably high.

Monte Carlo methods were used to construct lognormal distributions of endotoxin concentrations, $C_{\;\mathrm{Endo}\;ij}$, for each worker, i, in task, *j*, based on the statistical attributes of the measured concentration dataset. We assume a lognormal distribution for all tasks as short term exposures to air contaminants are often assumed to be lognormal [34]. The individual worker distributions in each task, $C_{\mathrm{Endo}\;ij}$, were aggregated to generate a single lognormal distribution, $C_{\;Endo\;j}$, for each task j = 1–5. Two parameter inputs were used to construct the 8-h TWA probability distributions ($TWA_{\;Endo\;s}$) across the selected exposure scenarios (*s*): (1) The lognormal distribution constructed for task specific endotoxin concentrations, $C_{\;Endo\;j}$, and (2) the observed set of durations, $t_{j}$, for each task j= 1–5, which were fixed by the scenario s and sum to eight.

To simulate an 8-h workday for a selected exposure scenario, the Monte Carlo model randomly selected a set of values from the task-specific concentration distributions $C_{\;\mathrm{Endo}\;j}$. The parameter representing the scenario specific set of durations spent in each task,$\;t_{js}$, was fixed in each scenario to ensure that rotation schedules were realistic and could be practically applied. For scenarios in which the $t_{1}-t_{5}$ does not sum to 8 h, we assume that the remainder of the day’s duration, $t_{6}$, is spent doing zero exposure tasks such as office work, lunch, or going offsite ($C_{\;6\;}=0$ for all scenarios *s*). For each exposure scenario, s, over the lognormal distribution of a random worker’s full 8-h workday, exposure to inhaled endotoxin is calculated as the following:

(1) $$TWA_{Endo\;s}=\overset{6}{\underset{j=1}{\sum }}\frac{C_{\;j}*t_{js}}{8}$$

for which $TWA_{Endo\;s\;}$is the concentration of endotoxin inhaled by a random worker in scenario s expressed in EU/m^3^. A simulation of 10,000 8-h workday concentrations was performed for each of the eights scenarios, s.

In addition to evaluating the exposures over an 8-h period, the concentration distributions, $C_{\;\mathrm{Endo}\;j},\;$enabled us to account for inherent variability in exposure measurements. Given this known variability of endotoxin exposure concentrations and the positive skewness of exposure distributions observed generally in workplaces, health risks may exist despite the presence of a sample set with few or limited high concentration data points [35]. To account for this, we calculated the probability of exceedance in each scenario defined as the probability that a randomly selected worker’s exposure would exceed the OEL on a randomly selected day. Exceedance was calculated from the within and between worker variances derived from the simulated concentration distributions across all tasks and considered acceptable if below 0.10 [36]. All Monte Carlo simulations were conducted in R, a free software environment for statistical computing (R-3.2.4 version, R Foundation for Statistical Computing, Vienna, Austria).

## 3. Results

### 3.1. Exposure Concentration by Task

In total, 62 air samples were collected—20 were area samples and 42 were personal samples collected from 13 workers. The duration of all tasks sampled varied between 28 and 250 min (Table 1). Task duration varied because of the intermittent supply of fecal sludge arriving at the facility. On days in which a large number of trucks dumped fecal sludge at the plant, the individual tasks were performed for longer durations of time compared to days in which the plant was operating under capacity because of the fewer trucks delivering fecal sludge.

**Figure 2:**
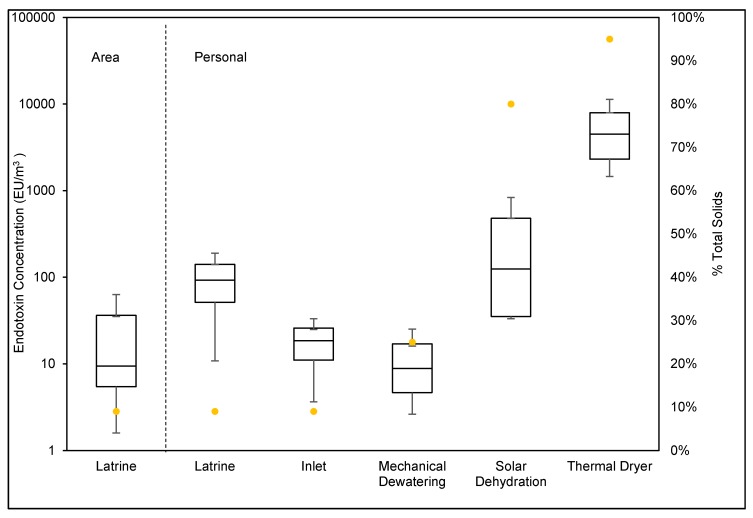
Endotoxin exposure by task and moisture content. The box plots show medians, 10th, 25th, 75th, and 90th percentiles (log base 10 scale) of endotoxin concentrations in the breathing zone of workers performing different tasks in the waste collection and resource recovery process. The dots indicate the total solid (TS) percentage of the sludge being handled in each task.

The range of endotoxin concentrations measured across personal task samples was between 2.6 EU/m^3^ to 9000 EU/m^3^. Concentrations of endotoxin varied significantly across the different work tasks (GSD = 11). The GM endotoxin exposure concentration was highest in the thermal drying task (3700 EU/m^3^) and lowest for the mechanical dewatering task (11 EU/m^3^). Endotoxin exposure increased with increasing total solid percentage of the fecal sludge being handled across the tasks. This trend is especially highlighted in the processes that occur at the plant during the three resource recovery tasks: mechanical dewatering, solar dehydration, and thermal drying (Figure 2).

### 3.2. Endotoxin Concentration in Personal vs. Area Samples:

Concentration of endotoxin varied significantly between personal samples and area samples in the pit latrine emptying task. Geometric mean levels of endotoxin were over six-fold higher in the personal samples than those in the area samples of the same pit latrine emptying task (75 vs. 12 EU/m^3^). The area samples exhibited a relatively high variability (GSD = 4.0) because of the varying distances from the latrines in which they were collected.

### 3.3. Exposure Scenario Analysis

Table 2 summarizes the output of a Monte Carlo model used to estimate the 8-h TWA endotoxin concentrations to which workers were exposed across seven different exposure scenarios.

The highest exposure scenario presented is the one in which the dryer is run for the maximum duration (of the durations observed during sampling campaign) while the lowest exposure scenario presented is one in which both the dryer and solar dehydration tasks are eliminated, and the inlet and mechanical dewatering tasks are completed for a minimal duration.

All scenarios that include the thermal dryer task (scenarios 1–2, Table 2) showed exposure concentrations with means and 95th percentile values considerably higher than scenarios that exclude the thermal dryer task (scenarios 3–8, Table 2). The 95th percentile concentration in all scenarios that included the thermal dryer task (scenarios 1–2) was between 12 and 24 times the OEL of 90 EU/m^3^ and the exceedance probabilities were between 0.74–0.75.

Of the scenarios in which the dryer task is eliminated, there is considerable variation in the means and 95th percentiles of the exposure concentrations. If the dryer is eliminated and a plant worker rotates through the other tasks for the maximum durations observed, the mean falls below the OEL but the 95th percentile and the probability of exceedance are beyond the acceptable limits. However, if the dryer is eliminated and a worker rotates through the other tasks for minimum durations, the mean and the 95th percentile fall below the 90 EU/m^3^ OEL but the probability of exceedance, at 0.21, remains above the 0.10 acceptable limit.

The lowest mean and 95th percentile exposure concentrations are seen in the two scenarios in which both the solar dehydration and thermal dryer tasks are eliminated (scenario 5–6). In both of these scenarios, the 95th percentile concentrations fall between three and 12 times below the OEL with exceedance probabilities of 0.04.

For the scenarios involving the waste collection team (scenarios 7–8), there is considerable difference between operating for minimum and maximum durations. In the scenario in which the waste collection team is operating for the maximum observed durations, the 95th percentile and the probability of exceedance fall above acceptable limits. In the scenario in which the waste collection team is operating for the minimum durations, the 95th percentile falls below the OEL, but the probability of exceedance is still above the acceptable limit.

## 4. Discussion

Our findings are consistent with a significant body of literature which shows that exposure varies significantly with task and type of waste handled [22,37].Unsurprisingly, the solar dehydration task and the thermal drying task, which exhibited the highest endotoxin concentrations, were also the tasks in which workers were handling drier material with a lower percentage of total solids. In the solar dehydration and thermal drying task, the relatively fecal sludge is handled and agitated in relatively enclosed spaces—the greenhouse and thermal dryer enclosure.

Though workers in the pit latrine, inlet, and mechanical dewatering tasks handled material with relatively low total solids, the measured endotoxin exposure concentrations were significantly higher in the latrine task. One possible reason for this could be related to the nature of the pit latrine emptying task itself which requires workers to come into closer proximity with sludge that they physically agitate during the fluidization process, trash removal, and hose guiding to facilitate pumping. The area sample data supports this hypothesis as concentrations from area samples of the latrine task are an order of magnitude lower than those of the personal samples for the same task. Similarly intermediate levels of endotoxin measured across the inlet and mechanical dewatering tasks may be explained as the combination of the relatively low total solids as well minimal manual agitation of the material compared to the pit latrine task.

The GM of 8-h TWA endotoxin exposure concentrations estimated for all exposure scenarios included in this study (range: 2.9–960 EU/m^3^) exhibited upper limits significantly higher than those observed in similar occupational environments (Agricultural workers in Colorado: 54 EU/m^3^; sewage treatment workers in the Netherlands: 27 EU/m^3^; solid waste workers in Korea: 2.2 EU/m^3^) but lower than occupational environments with the highest reported concentrations (Pig Farming: 1510 EU/ m^3^) [17,23,38,39].

The exceedance probability, or the probability of a randomly selected worker’s mean exposure exceeding the OEL, was in excess of the 0.10 recommended level for all scenarios except for the scenario in which both the greenhouse task and the thermal dryer task are eliminated. This calls for a prioritization of controls and process re-designs that limit or eliminate worker exposure in these two tasks. Effective elimination of these two tasks can reduce both 95th percentile TWA concentration as well as the probability of exceedance to below 0.10. While the 95th percentile exposure concentrations of scenarios 7 and 9 are below the OEL. In both of these scenarios the probability of exceedance is above 0.10. This demonstrates the value of a metric such as exceedance, which accounts for sample and distribution features such as variability over days and workers before deeming an exposure compliant with an OEL.

A core strength of this study is the use of conservative exposure sampling and characterization methods that are generally protective of worker health—personal task-based sampling and stochastic modeling of exposure concentrations. 

Unlike the area sampling methods that are typically used in occupational exposure sampling studies, personal sampling methods provide exposure estimates that account for the task being performed, the distance from the hazard source, and other human factors. A personal sampling strategy also enabled us to calculate the probability of exceedance and overexposure through within and between person variance analysis.

Task-based sampling methods provide a more refined evaluation of exposure variability compared to assessing exposures using continuous 8-h time weighted averages across different tasks. Furthermore, when resources to control exposures are limited, task-based exposure concentration differences are useful for prioritizing interventions.

Using a Monte Carlo modeling framework allowed us to simulate and subsequently characterize exposure concentrations despite a relatively small number of collected samples and a contaminant, endotoxin, which is known to exhibit exposure variability depending on the various environmental and occupational factors [32]. This variability in measured endotoxin concentrations is apparent in our sample set, particularly in those collected from the inlet, mechanical dewatering, and solar dehydration tasks. Failing to account for this variability could substantially limit the validity of conclusions. In essence, large health risks may exist despite a sample set with relatively low measured exposure concentrations [35,40]. While repeated measurements are the most effective way to drive down this uncertainty, collecting large amounts of samples may be prohibitively expensive, especially in resource strapped contexts. In this study we combine empirical measurements with modeling, a valuable approach for low-resource contexts.

There are several limitations to this study. For one, because of resource limitations, we were unable to sample endotoxin exposures to workers across different seasons. Seasonality has been reported as a significant determinant of endotoxin exposure levels in both occupational and background environmental contexts [41]. In this study we sampled during Rwanda’s dry season but more research is needed to understand how the growth of bacteria, release of endotoxin, and exposure to airborne endotoxin are affected by seasonal changes in factors such as temperature, wind, and humidity.

Because the area samples were collected during a different year than personal air samples, another limitation is that the reported magnitude in the difference between area and personal samples may be biased by factors that were not quantified such as differences in meteorological factors or factors related to sample storage and processing.

Finally, though differences in exposures have been displayed here, further work is necessary to establish the association between exposure to endotoxin and respiratory symptoms. Factors such as variable breathing rates related to the strenuousness of different tasks may influence final exposure dose and the resulting health effects.

Because of scope limitations of this study, we did not collect data on the background endotoxin concentration levels outside of the waste-to-fuel processes. Thus, further study is needed to evaluate the relative risk of workers in the process studied compared to non-occupational exposures of residents living in the city of Kigali.

To the authors’ knowledge, this is the first study in the published literature to report airborne endotoxin concentrations or exposure assessment data related to fecal sludge management and resource recovery sanitation systems. The lack of exposure data for workers in fecal sludge management and reuse processes is likely due to the novelty of such processes as well as limited capacity for sample collection and analysis in the low-resource settings in which they are deployed.

## 5. Conclusions

Resource recovery sanitation systems can provide immense societal benefits because of their impact on disease reduction and environmental protection. However, little is known about the potential occupational health effects these systems may pose on workers. As our study shows, endotoxin exposure for workers doing certain tasks in the fecal sludge collection and resource recovery process is significant and deserves more study. As new resource recovery systems emerge, incorporating occupational exposure considerations into the design and optimization of emerging systems is necessary to safeguard public health for communities and workers alike. This study outlines an exposure assessment method combining workplace sampling techniques and stochastic modeling. We provide both a methodological approach for use in further occupational exposure studies as well as preliminary insights on endotoxin exposures to workers in a municipal fecal sludge-to-fuel process.

The results of this study can be used to inform the design and optimization of emerging waste collection and resource recovery processes. A particular emphasis should be placed on controlling endotoxin exposure during tasks that require workers to be in close proximity with sludge or in working with material with a high percentage of total solids whose dust can be easily aerosolized. A combination of engineered controls, process changes, and personal protective equipment (PPE) can be employed. Examples of engineering controls may include improved pumping equipment that maximize the distance between the sludge containing pit and operator, automated tilling equipment to eliminate the need for manual agitation of dry sludge in the greenhouse, and an automated conveyer belt for loading dry sludge into the thermal dryer and final storage bags. While process changes are preferred and engineering controls are preferred, the use of personal protective equipment (PPE) may be necessary in low-resource contexts where there are constraints to implementing equipment and infrastructure changes. If personal protective equipment is used, administrative controls would need to be set in place to ensure that respirators are properly fit tested, stored, and routinely replaced. Hazard communication strategies should also promote general workplace hygiene practices aimed at reducing occupational exposures.

## Figures and Tables

**Table 1:** Endotoxin exposure concentrations and sample durations by task type.

Sample Type	Task	*n*	Endotoxin (EU/m^3^)			Duration (min)	
			GM (GSD)	AM (SD)	Range	AM (SD)	Range
Area	Latrine	20	12 (4.0)	35 (76)	1.6–350	190 (67)	109–394
Personal	Latrine	11	75 (2.4)	97 (61)	11–200	130 (57)	73–251
Personal	Inlet	2	11 (4.8)	18 (21)	3.6–33	75 (37)	48–101
Personal	Mech. Dewatering	10	11 (3.5)	29 (61)	2.6–200	96 (44)	31–167
Personal	Solar Dehydration	10	140 (4.8)	450 (820)	33–2700	53 (19)	28–95
Personal	Thermal Drying	9	3700 (2.0)	4600 (820)	1500–9000	80 (24)	48–105

**Table 2:** Modeled probability distributions 8-h TWA exposure scenarios.

Exposure Scenario *s*	Description, Task Duration $t_{js}$	Geometric Mean (GSD) $TWA_{Endo\;s}$	Arithmetic Mean (SD) $TWA_{Endo\;s}$	95th Percentile $\;TWA_{Endo\;s}$	Probability of Exceedance γ
1	-Dryer run for max duration ($t_{5\;}=\max\left(t_{5\;}\right)$) -Average time spent in other plant tasks $\mu \;\left(t_{2},t_{3\;},t_{4}\right)$	960 (1.6)	1091 (580)	2200	0.75
2	-Dryer run for min duration ($\;t_{5\;}=\min\left(t_{5\;}\right)$) -Average duration spent in other plant tasks $\mu \;\left(t_{2},t_{3\;},t_{4}\right)$	460 (1.6)	520 (270)	1040	0.74
3	-Dryer not run ($\;t_{5\;}=0$) -Max duration spent in other plant tasks max$\left(t_{2},t_{3\;},t_{4}\right)$	54 (2.0)	71 (65)	185	0.40
4	-Dryer not run ($\;t_{5\;}=0$) -Min. duration spent in other plant tasks max$\left(t_{2},t_{3\;},t_{4}\right)$	15 (2.0)	21 (19)	53	0.21
5	-Dryer and solar dehydration not done. ($\;t_{4\;,}t_{5\;}=0$) -Max. duration spent in other plant tasks max$\;\left(t_{2},t_{3\;}\right)$	11 (1.9)	13 (11)	26	0.04
6	-Dryer and solar dehydration not done. ($\;t_{4\;,}t_{5\;}=0$) -Min. duration spent in other plant tasks min$\left(t_{2},t_{3\;}\right)$	2.9 (1.8)	3.5 (2.7)	7.8	0.04
7	-Latrine emptying only for maximum duration. ($\;t_{1}=\max\left(\;t_{1}\right)$) -No other tasks done ($\;t_{2},t_{3\;},t_{4\;,}t_{5\;}=0$)	24 (1.4)	26 (8.6)	42	0.11
